# The Cytotoxicity and Nephroprotective Activity of the Ethanol Extracts of *Angelica keiskei* Koidzumi Stems and Leaves against the NAPQI-Induced Human Embryonic Kidney (HEK293) Cell Line

**DOI:** 10.1155/2021/6458265

**Published:** 2021-11-23

**Authors:** Riezki Amalia, Diah Lia Aulifa, Dichy Nuryadin Zain, Anisa Pebiansyah, Jutti Levita

**Affiliations:** ^1^Department of Pharmacology and Clinical Pharmacy, Faculty of Pharmacy, Universitas Padjadjaran, Sumedang, Bandung 45363, Indonesia; ^2^Department of Pharmaceutical Analysis and Medicinal Chemistry, Faculty of Pharmacy, Universitas Padjadjaran, Sumedang, Bandung 45363, Indonesia; ^3^Bakti Tunas Husada School of Health Sciences, Tasikmalaya 461152, Indonesia

## Abstract

**Materials and Methods:**

*A*. *keiskei* Koidzumi plant was collected from Mount Rinjani, Lombok, Indonesia, and was identified at the School of Biology Sciences and Technology, Bandung Institute of Technology, Indonesia. Extraction of the stems (ASE) and leaves (ALE) was performed by employing ethanol 70% for 3 × 24 h at 26°C. The cytotoxicity study of the extracts was assessed using the water-soluble tetrazolium salt-8 (WST-8) reagent on the HEK293 cell line, while the nephroprotective activity assay was determined on the NAPQI-induced HEK293 cell line.

**Results:**

The WST-8 assay showed that the cytotoxicity IC_50_ of ASE = 2322 *μ*g/mL and IC_50_ of ALE = 2283 *μ*g/mL. The nephroprotective activity assay revealed that ASE possesses nephroprotective activity against the NAPQI-induced HEK293 cell line at 1161 *μ*g/mL, while ALE does not show the nephroprotective activity.

**Conclusion:**

Taken together, lower concentrations of ASE and ALE (<2000 *μ*g/mL) are not toxic to the HEK293 cell line, and only ASE indicates the activity to protect the HEK293 cell line against NAPQI damage. This Japanese celery could be further explored for its potential as a plant-based nephroprotective drug.

## 1. Introduction

Kidney injury arises when its physiological functions, detoxification, and excretion do not perform properly. This dysfunction is usually caused by exogenous, e.g., drugs (anti-inflammatories, antibiotics, and chemotherapeutics), or endogenous toxicants (oxidative stress products such as reactive oxygen species due to intracellular catabolism and activation of oxidative enzymes, e.g., superoxide dismutase) [1–4]. Acetaminophen, an anti-inflammatory drug, when taken in excess, can lead to hepatotoxicity and nephrotoxicity due to glutathione (GSH) depletion in the liver; hence, the reactive metabolite of acetaminophen, N-acetyl-p-benzoquinone imine (NAPQI), is not conjugated [5]. Nephrotoxicity is indicated by changes in glomerular filtration rate (normal GFR in young adults is 120 mL/minute), proximal tubular cell toxicity, inflammation, etc., and the enzymes present in tubular epithelial cells leak into the urine and can be determined as nephrotoxic biomarkers [1]. Such biomarkers, e.g., neutrophil gelatinase-associated lipocalin (NGAL), kidney injury molecule-1 (KIM-1), and calprotectin, are used as an early diagnosis of acute and chronic kidney injury [6, 7].

Many natural products with antioxidant properties have been reported to have advantageous effects in the remedy of nephrotoxicity [8]. *Angelica keiskei* Koidzumi or ashitaba (a Japanese word which means tomorrow leaf) has been believed in improving health, particularly protecting the liver and kidney system, and achieving longevity. This plant has shown many pharmacology activities; among them is as antioxidants [9–11].

In this study, we investigated the cytotoxicity and nephroprotective activity of the ethanol extract of *A*. *keiskei* Koidzumi on the N-acetyl-p-benzoquinone imine (NAPQI) induced human embryonic kidney (HEK293) cell line. NAPQI was chosen as the kidney injury inducer because this reactive metabolite of acetaminophen binds covalently to the sulfhydryl groups of renal proteins and leads to the damage of proximal tubules. It also initiates the apoptosis process involving the activation of caspase-9 and caspase-3 by generating free radicals [12].

## 2. Materials and Methods

### 2.1. Plant Materials

**Figure 1:**
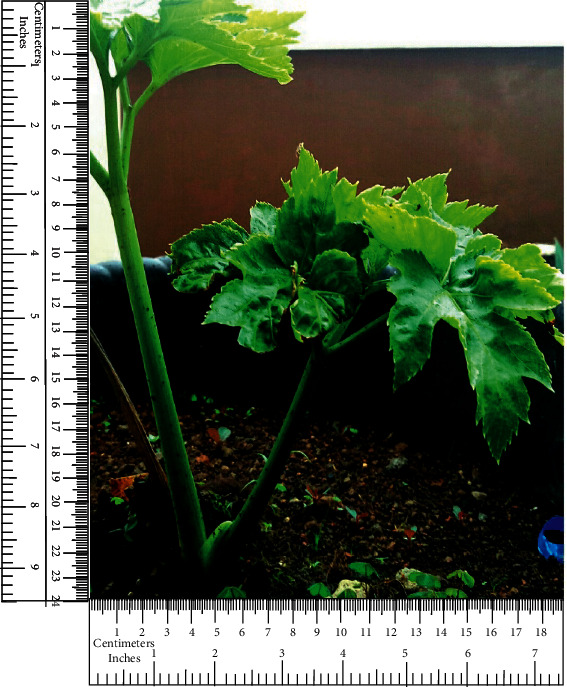
*Angelica keiskei* Koidzumi or Japanese celery.

The *A*. *keiskei* Koidzumi plant (Figure 1) was collected from Mount Rinjani, Lombok, and was taxonomically identified at the School of Biology Sciences and Technology, Bandung Institute of Technology, Indonesia. The stems and leaves were washed under tap water to remove dirt and soil and were dried in the glasshouse for 2 days. The dried plants were ground to pass a mesh-60 sieve and kept for further use.

### 2.2. Materials and Chemicals

The materials used were HEK293 cell line (ATCC^®^ CRL-1573™), a collection of the Cell and Molecular Biology Laboratory, Faculty of Pharmacy, Universitas Padjadjaran. Chemicals were N-acetyl-p-benzoquinon**e** imine (NAPQI) (CAS reg. no. 50700-49-7, Cayman Chemical, Ann Arbor, USA), quercetin (CAS reg. no. 117-39-5, Sigma-Aldrich, Saint Louis, USA), Cell Counting Kit-8: WST (product code: CK04-11, Dojindo Europe), Dulbecco's Modified Eagle Medium (Gibco), and penicillin-streptomycin (Gibco^™^ 670087).

### 2.3. Instruments

Instruments used were an evaporator Rotavapor RV 10 Digital V connected to heating bath HB digital and RV 10.1 set of glassware vertical (IKA Id. no. 0010004799), chemical glassware (Iwaki Pyrex), autoclave (all American type 75X), Biological Safety Cabinet (BSC) type-2, microplate reader (Infinite M200 Pro, Tecan), microplate reader filter 450–490 nm, inverted microscope (Zeiss), sterilized 96-well microplates, multichannel pipettes (8 or 12 channels: 10–100 *μ*L), CO_2_ incubator (Heracell VIOS 250i, Thermo Scientific), and hemocytometer (cell counter).

### 2.4. Extraction

Extraction of the stems (ASE) and leaves (ALE) was performed by employing ethanol 70% for 3 × 24 h at 26°C. The extracts were then evaporated at 60°C to remove the solvent, freeze-dried to obtain the dry extracts, and were stored at 4°C until used.

### 2.5. Cytotoxicity Assay of the Extracts

The cytotoxicity of ASE and ALE towards the HEK293 cell line was assessed using the water-soluble tetrazolium salt-8 (WST-8) reagent. WST-8 (2-(2-methoxy-4-nitrophenyl)-3-(4-nitrophenyl)-5-(2,4-disulfophenyl)-2H tetrazolium, monosodium salt) is highly stable and utilized in Cell Counting Kit-8 (CCK-8) [13]. ASE and ALE solutions were prepared by dissolving 32 mg extract in 1 mL of 1% DMSO in a culture medium. The solution was serial-diluted to concentrations of 160 *μ*g/mL, 320 *μ*g/mL, 640 *μ*g/mL, 1280 *μ*g/mL, 2560 *μ*g/mL, and 5120 *μ*g/mL in DMSO 1%. The percentage growth inhibition was calculated using the following formula [14]:

(1) $$\begin{array}{c} \text{growth inhibition}\left(\%\right)=\left(1-\frac{\text{absorbance sample}}{\text{absorbance control}}\right)\times 100\%. \end{array}$$

IC_50_ was calculated using GraphPad Prism 8.0.2.

The cytotoxicity of quercetin and NAPQI was assessed using the same procedure. Quercetin and NAPQI solutions were prepared as follows: accurately weighed quercetin (605 *μ*g) and NAPQI (500 *μ*g) were each dissolved in 1 mL of 1% DMSO in the culture medium. The solution was serial-diluted to concentrations of 15.13 *μ*g/mL, 30.25 *μ*g/mL, 60.5 *μ*g/mL, 121 *μ*g/mL, and 242 *μ*g/mL for quercetin and 15.63 *μ*g/mL, 31.25 *μ*g/mL, 62.5 *μ*g/mL, 125 *μ*g/mL, and 250 *μ*g/mL for NAPQI, respectively, in DMSO 1%.

### 2.6. Nephroprotective Activity Assay

The nephroprotective activity assay was determined on the NAPQI-induced HEK293 cell line by adopting the method of Nafiu and coworkers [14] with modifications. The assay was performed on six groups for each ASE and ALE, which were (1) normal control; (2) negative control (NAPQI: 75.00 *μ*g/mL); (3) positive control (quercetin: 45.68 *μ*g/mL); (4) positive control II (quercetin: 91.36 *μ*g/mL); (5) ASE or ALE concentration I; and (6) ASE or ALE concentration II. To each well of the microplate, 50 *μ*L of diluted HEK293 cells (approximately 5 × 10^3^ cells) was added. Cultures were maintained at 37°C in a humidified atmosphere of a 5% CO_2_ incubator. After 24 h, the supernatant was discarded, and the monolayer of cells was washed with DMEM, and 50 *μ*L of ASE in 1% DMSO (1161 *μ*g/mL and 2322 *μ*g/mL, respectively, in three replicates) and ALE (1141 *μ*g/mL and 2283 *μ*g/mL, respectively, in three replicates) was added. The microplate was incubated at 37°C in 5% CO_2_ for 1 h, followed by the addition of 50 *μ*L of NAPQI 75 *μ*g/mL solution. After 24 h of incubation, the medium was flicked off, and 10 *μ*L of WST-8 reagent was added. The mixture in the microplate was incubated for 2 h at 37°C in 5% CO_2_. Following this, a volume of 100 *μ*L HCl was added to each well to stop the reaction, and the absorbance of the mixture was measured at 450 nm using a microplate reader.

### 2.7. Statistical Analysis

Results are presented as the mean and standard deviation of replicate experiments. One-way ANOVA and Duncan's multiple range tests were used to determine significant differences (*p* < 0.05 was considered statistically significant).

## 3. Results

**Figure 2:**
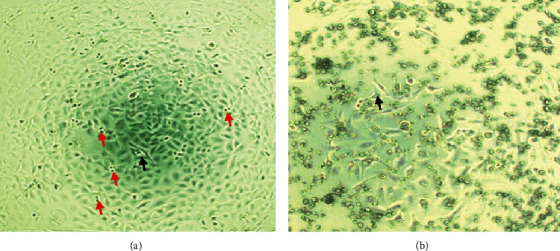
Microphotograph of (a) untreated HEK293 cell line and (b) *A*. *keiskei*-treated HEK293 cell line. Magnification: 200x. The black arrow indicates the dendritic process, and the red arrow indicates binucleate cells.

As depicted in the photomicrographs (Figure 2), the untreated HEK293 and the *A*. *keiskei*-treated HEK293 cell lines show only slight differences. The untreated cells could be seen located in a wider space, and some indicated dendritic processes to reach their neighboring cells. Some of the cells are binucleate that indicated proliferation. The *A*. *keiskei*-treated HEK293 cells revealed lesser dendritic processes and the absence of binucleate.

**Figure 3:**
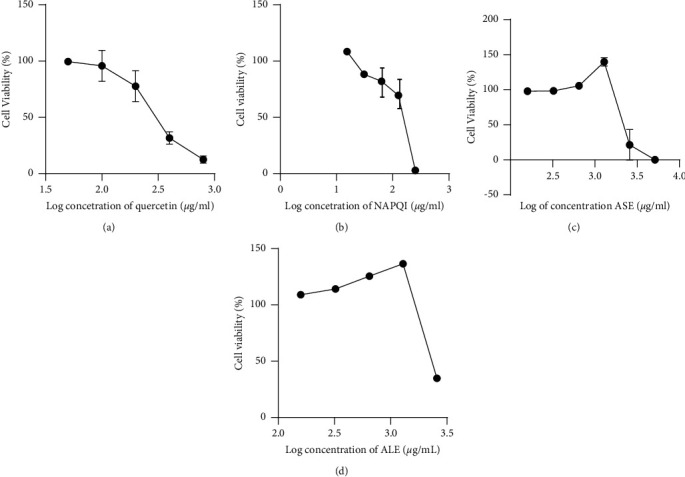
Cytotoxicity of quercetin (a) (IC_50_ = 91.35 *μ*g/mL or 0.302 mM), NAPQI (b) (IC_50_ = 163.19 *μ*g/mL or 1.093 mM), the ethanol extract of (c) *A*. *keiskei* stem (ASE; IC_50_ = 2322 *μ*g/mL), and (d) *A*. *keiskei* leaves (ALE; IC_50_ = 2283 *μ*g/mL) on HEK293 cell lines.

Furthermore, the nephroprotective activity assay (Figure 3) reveals that ASE possesses nephroprotective activity on the NAPQI-induced HEK293 cell line at 1161 *μ*g/mL, while ALE does not.

## 4. Discussion

N-Acetyl-p-benzoquinone imine (NAPQI) is a reactive metabolite of acetaminophen (paracetamol), which has been confirmed for its damage effect on the liver and kidney due to glutathione depletion. NAPQI cytotoxicity has been studied in lymphoblastoid cell lines derived from Caucasian-American, African-American, and Han Chinese-American healthy subjects. Average NAPQI IC_50_ for those cell lines was 6.5 ± 4.5 *μ*M. Lower concentrations of NAPQI resulted in a proliferation increase in many of the cell lines [5]. In the body, NAPQI binds to selenium protein and glutamine synthetase at the S3 segment of the proximal tubule, which causes an increase of xanthine oxidase (XOD) activity; thus, the production of reactive oxygen species (ROS) is also enhanced [14, 15]. Excess ROS production and its exposure to the kidney leads to oxidative stress and is followed by nephropathy and eventually kidney failure [8, 16]. In this study, we investigated the cytotoxicity and nephroprotective activity of the ethanol extract of *A*. *keiskei* Koidzumi on the NAPQI-induced HEK293 cell line.

**Figure 4:**
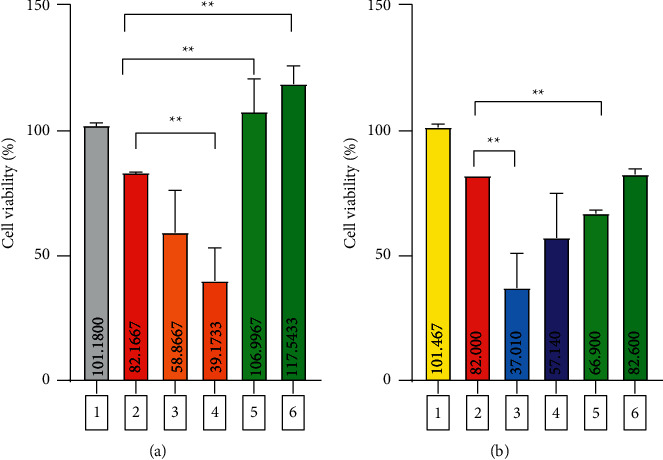
The nephroprotective activity of ASE (a) and ALE (b) on the NAPQI-induced HEK293 cell line. 1: normal control group; 2: negative control group; 3-4: positive control group (quercetin: 45.68 *μ*g/mL and 91.36 *μ*g/mL); 5-6: assayed extract group. ^*∗∗*^ indicates a significant difference with the negative control group (*p* < 0.05).

In the NAPQI-induced HEK293 cell line (the negative control group), only approximately 82% of the cells were viable (Figure 3), compared to the extract-treated groups (Figure 3). The result obtained in our study is comparable with the previously reported result given by Kwon and coworkers. The chlorophyll-rich methanol extract of *A*. *keiskei* planted in Korea possesses a strong antioxidant activity [11]. The radical scavenging effect of the leaf extract was stronger than that of the extract of the stem [17]. This antioxidant activity is predicted caused by certain phytoconstituents contained in the plant. However, various secondary metabolites, e.g., flavonoids, chalcones, coumarins, phenolics, acetylenes, and terpenes, have been identified in different parts of *A*. *keiskei* [18, 19]. Unexpectedly, quercetin (IC_50_ = 91.35 *μ*g/mL or 0.302 mM), a well-known flavonoid, in the concentration of 45.68 *μ*g/mL and 91.36 *μ*g/mL reduced the viability of the HEK293 cell line (Figures 4 and 3).

Our result is in accordance with that of Dugan [16]. Dugan had pretreated HEK293 cells with 10–100 *μ*M concentrations of quercetin. Then, the cells were 24-hour toxicity-induced with 30 *μ*M of cadmium chloride. It was suggested that higher concentrations of quercetin (>10 *μ*M) could stimulate an increase in cell death. In higher concentrations, quercetin was predicted to enhance the toxic effect of cadmium in HEK293 cells through JNK phosphorylation [16]. Nevertheless, in another study on cultured granulosa cells from chicken ovarian follicles, quercetin confirmed the potential in preventing cadmium-induced cytotoxicity. This protective activity of quercetin was predicted by diminishing lipid peroxidation, enhancing the antioxidant status within the cells, and inhibiting apoptosis [20].

A study on the effect of chalcone derivatives on D-galactosamine/lipopolysaccharide-induced liver failure in mice has confirmed a strong hepatoprotective activity of those compounds [21]. Chalcones have been disclosed as inhibitors of the synthesis of triglycerides. These open-ring flavonoids also work as activating factors of hepatic stellate cells and extracellular matrix deposition; thus, they could be used as liver-protective drugs [22]. Moreover, several flavonoids have revealed kidney-protective activities against many nephrotoxic agents, such as lipopolysaccharide, gentamycin, lead, or cadmium. In studies in murine models of chronic kidney disease, flavonoids significantly impaired kidney function [23]. A chalcone derivate has been reported which could significantly decrease the key markers for renal and cardiac dysfunction in diabetic mice [24].

## 5. Conclusions

The lower concentration of the ethanol extract of *Angelica keiskei* Koidzumi stems and leaves is not toxic to the human embryonic kidney (HEK)293 cell line, and only the stem extract protects the HEK293 cell line against N-acetyl-*p*-benzoquinone imine (NAPQI) damage. Chalcones and flavonoids contained in the stem extract might play a key role in this protective activity by scavenging the reactive oxygen species. This Japanese celery could be further explored for its potential as a plant-based nephroprotective drug.

## Data Availability

The data used to support the findings are available on request from the corresponding author.
